# Elevated subgingival temperature infers high bacterial pathogen counts in severe periodontitis

**DOI:** 10.1002/cre2.891

**Published:** 2024-05-06

**Authors:** Thomas E. Rams, Jørgen Slots

**Affiliations:** ^1^ Department of Periodontology and Oral Implantology Temple University School of Dentistry Philadelphia Pennsylvania USA; ^2^ Division of Periodontology and Diagnostic Sciences University of Southern California School of Dentistry Los Angeles California USA

**Keywords:** inflammation, periodontal diagnosis, periodontitis, *Porphyromonas gingivalis*

## Abstract

**Objectives:**

Periodontal inflammation may be assessed by bleeding on probing and subgingival temperature. This pilot study evaluated the intrapatient relationship between subgingival temperature and selected bacterial groups/species in deep periodontal pockets with bleeding on probing.

**Materials and Methods:**

In each of eight adults, an electronic temperature probe identified three “hot” pockets with elevated subgingival temperature and three “cool” pockets with normal subgingival temperature among premolars/molars with 6‒10 mm probing depths and bleeding on probing. Microbial samples collected separately from the hot and cool periodontal pockets were cultured for selected periodontal pathogens.

**Results:**

Hot compared to cool periodontal pockets revealed significantly higher absolute and normalized subgingival temperatures and yielded higher mean proportions of *Porphyromonas gingivalis* (10.2% for hot vs. 2.5% for cool, *p* = 0.030) and total red/orange complex periodontal pathogens (48.0% for hot vs. 24.6% for cool, *p* = 0.012).

**Conclusions:**

Hot versus cool deep periodontal pockets harbored significantly higher levels of major periodontal pathogens. Subgingival temperature measurements may potentially be useful to assess risk of periodontitis progression and the efficacy of periodontal therapy.

## 1 INTRODUCTION

Treatment of severe periodontitis remains a challenge in dentistry (Slots, 2022). Periodontitis patients typically receive conventional (mechanical) therapy which may not always control the extraordinarily complex mixture of pathogenic bacteria and active herpesviruses existing within deep periodontal pockets, narrow furcation sites, and inflamed gingiva (Rams & Slots, 2023). Also, because the initial onset of periodontal breakdown tends to induce major tissue destruction (Slots & Rams, 2023), periodontics may preferably employ preemptive therapy which uses clinical and/or laboratory tests to identify high‐risk patients and guide effective treatment before impending disease progression.

Periodontal disease is currently assessed by clinical, radiographic, and biological parameters, each of which has advantages and limitations. Periodontal pocket depth and alveolar bone height measure past periodontal destruction but not necessarily the current or future rate of periodontal breakdown (Armitage, 2013). Absence of gingival bleeding on probing (Gonzalez et al., 2015) and the presence of radiographic crestal alveolar lamina dura (Rams et al., 2018a) are valuable low‐risk indicators for periodontal breakdown (high sensitivity), but periodontal sites can show bleeding on probing and undetectable crestal alveolar lamina dura and still remain disease stable long‐term (low specificity). Microbiological monitoring (Rams & Slots, 2023; Rams et al., 1996; Saygun et al., 2011) and gingival fluid testing for bony and connective tissue breakdown products (Slots, 2017) may help identify risk of active periodontitis, but available tests tend to be cumbersome, relatively expensive, and not uniformly predictive. Computerized algorithms in lieu of routine checklists to interpret a series of diagnostic tests might improve the probabilistic risk assessment of destructive periodontal disease, but the components and critical values for such algorithms remain to be established.

Because elevated temperature is a classic feature of inflammation, periodontal pocket temperature may supply diagnostically relevant insight on periodontal disease status. van der Velden (2017) proposed that elevated subgingival temperature correlates with greater pocket depth and increased odds for periodontal breakdown. Bacterial pathogens (Haffajee et al. 1992c; van der Velden, 2017) and active herpesviruses (Slots, 2015) within periodontal pockets and inflamed gingiva are assumed to induce the periodontal elevated temperature. However, it is not clear if microbiological differences detected in “hot” and “cool” periodontal sites between patients (Haffajee et al., 1992c) also occur at such sites within patients. The present study examined the intra‐patient relationship between deep periodontal pocket temperature and cultivable subgingival proportions of major periodontal pathogens.

## 2 MATERIALS AND METHODS

### 2.1 Patients

A cross‐sectional pilot study included four male and four female adults, who were systemically healthy and nonsmokers with a mean age of 44.9 years (standard deviation [SD] = 5.6 years) and localized to generalized Stage III/Grade B periodontitis (severe) (Tonetti et al., 2018). The present study was carried out 4‒6 weeks after completion of oral hygiene instructions and nonsurgical root debridement (Kwon et al., 2021) and conducted in accordance with the Declaration of Helsinki, with approval of the Temple University Institutional Review Board, and the patients signed informed consent.

### 2.2 Clinical examinations

A calibrated periodontist (coauthor Thomas E. Rams) performed the temperature measurements and clinical evaluations (American Academy of Periodontology, 2000). An electronic temperature probe (PerioTemp Probe System, ABIODENT Inc.) measured absolute subgingival and sublingual site temperatures and determined normalized differential values of subgingival temperature relative to sublingual temperature. The device, cleared for clinical use by the United States Food and Drug Administration, has a computer‐linked thermocouple embedded inside a plastic periodontal probe tip with dimensions and shape similar to a Michigan‐O probe (Kung et al., 1990), a foot‐activated switch, and a print‐out of measured values (Kung et al., 1990). As a proxy for body core temperature, the probe tip placed for 5 s under the most posterior and medial part of the tongue, measured sublingual temperature to the nearest 0.01°C. Subgingival temperature was determined by gently advancing the probe tip to full pocket depth at six sites of each study tooth. The temperature probe provided a normalized subgingival temperature differential by subtracting the patient's sublingual temperature from the subgingival temperature (Kung et al., 1990). The temperature probe designated the subgingival temperature as either “hot” (red light) or “cool” (green light) after taking into account the normal warmer posterior versus cooler anterior teeth and warmer maxillary versus cooler mandibular teeth (Kung et al., 1990) and the variability in subgingival temperature among periodontally healthy individuals (Haffajee et al., 1992a). The tooth‐specific temperature threshold values are published elsewhere (Haffajee et al., 1992a; Kung et al., 1990) and were utilized in prior studies (Haffajee et al., 1992a, 1992b, 1992c;  Kung et al., 1990; Maiden et al., 1998; Niederman et al., 1995; Rams & Slots, 2023) as well as in the present study. The reproducibility of temperature measurements was determined in replicate readings taken 2 min apart at 18 sublingual and 55 subgingival sites. Bleeding on probing was assessed within 30 s after the removal of the temperature probe from subgingival sites. A Michigan‐O probe assessed periodontal pocket depth and a Nabers probe furcation involvement.

### 2.3 Microbiological examination

Subgingival specimens were collected with paper points from periodontitis patients who each contributed samples from three hot and three cool premolar/molar pockets with a probing depth of 6‒10 mm and bleeding on probing. Each periodontal sample originated from separate teeth and interproximal surfaces and was transported in VMGA III (Dahlén et al., 1993) within 24 h to the oral microbiology laboratory at the University of Southern California School of Dentistry, Los Angeles, California, USA, which was licensed for high‐complexity bacteriology by the California Department of Public Health and the United States Centers for Medicare and Medicaid Services. Enriched Brucella blood agar comprised the nonselective culture medium, Hammond's selective medium was used for *Campylobacter* isolation (Rams et al., 1993b), and TSBV agar for *Aggregatibacter actinomycetemcomitans* (Slots, 1982). *Porphyromonas gingivalis, Tannerella forsythia, Campylobacter* species, *Fusobacterium nucleatum*, *Parvimonas micra, Prevotella intermedia/nigrescens*, and *A. actinomycetemcomitans* were routinely identified using established methods (Rams & Slots, 2023; Rams et al., 1996, 2016, 2018b, 2023), and the percentage of each study species was calculated. Phase‐contrast microscopy enumerated the proportional distribution of spirochetes and motile rods among total subgingival morphotypes (Slots et al., 1979). The microbiology procedures were performed blinded to the clinical status of the patients and the periodontal study sites.

### 2.4 Data analysis

Periodontal bacteria were grouped into Socransky's clusters (red complex, orange complex, and other species) (Socransky et al., 1998). Red/orange complex species proportions were determined for each patient and their mean value across all patients. The nonparametric Wilcoxon matched pairs signed‐rank test and the McNemar's nonparametric test with Yates’ continuity correction assessed clinical and microbiological differences between hot and cool periodontal sites. A *p* ≤ 0.05 was required for statistical significance. Data analysis was performed using the STATA/SE 16.1 for Windows 64‐bit statistical software package (StataCorp PL).

## 3 RESULTS

### 3.1 Clinical findings

Periodontal pockets with elevated temperature were identified on 14 maxillary and 10 mandibular premolars and molars and periodontal pockets with nonelevated temperature on 6 maxillary and 17 mandibular premolars and molars. Hot periodontal pockets had an average depth of 7.9 ± 1.3 (SD) mm (range 6‒10 mm) per patient, and cool periodontal pockets an average depth of 7.5 mm ± 1.3 (SD) (range 6‒10 mm) (*p* = 0.444). Replicate temperature measurements averaged 0.06 ± 0.05 (SD) °C for subgingival temperature and 0.1 ± 0.08 (SD) °C for sublingual temperature, indicating high reproducibility in the temperature measurements.

Hot periodontal pockets per patient showed significantly higher absolute temperatures (averaging 37.03°C, range 36.38–38.31°C) than cool periodontal pockets (averaging 36.58°C, range 34.34–38.0°C) (*p* = 0.031). Hot periodontal pockets per patient also revealed higher normalized (compared to sublingual temperature) subgingival temperatures (mean temperature differential of −0.21°C; range −0.60°C to + 0.35°C) than cool periodontal pockets (mean temperature differential of −0.60°C; range −2.61°C to −0.11°C) (*p* = 0.029).

### 3.2 Microbiological findings

Table 1 shows the microbiological findings. *Porphyromonas gingivalis* exhibited significantly higher proportions per patient in hot (10.2%) than in cool (2.5%) periodontal pockets (*p* = 0.030). Total subgingival proportions of red/orange complex species per patient averaged 48.0% in hot and 24.6% in cool periodontal pockets (*p* = 0.012), and the difference in red/orange complex bacteria remained statistically significant even after excluding the proportions for *P. gingivalis* (37.8% in hot vs. 18.7% in cool pockets, *p* = 0.012). *Parvimonas micra* and *Campylobacter* species tended to be higher in hot periodontal pockets but did not reach statistical significance. The remaining test bacteria did not differ significantly between hot and cool periodontal pockets.

**Table 1:** Periodontal pathogens in hot and cool deep periodontal pockets with bleeding on probing.

	No. (%) positive patients			Subgingival proportions (mean % ± SD)		
Subgingival species	hot sites	cool sites	*p*‐Value	Hot sites	Cool sites	*p*‐Value
Total anaerobic counts/mL (log_10_)	8 (100)	8 (100)	NS	8.3 ± 8.0	8.3 ± 7.9	NS
Red complex species:						
* Porphyromonas gingivalis*	5 (62.5)	3 (37.5)	NS	10.2 ± 9.8	2.5 ± 4.6	0.030
* Tannerella forsythia*	2 (25.0)	3 (37.5)	NS	0.2 ± 0.5	0.2 ± 0.4	NS
Orange complex species:						
* Campylobacter* species	8 (100)	8 (100)	NS	11.7 ± 24.6	2.3 ± 2.3	NS
* Fusobacterium nucleatum*	7 (87.5)	7 (87.5)	NS	8.9 ± 6.6	9.2 ± 15.9	NS
* Parvimonas micra*	7 (87.5)	7 (87.5)	NS	12.3 ± 16.5	5.5 ± 4.6	NS
* Prevotella intermedia/nigrescens*	4 (50.0)	6 (75.0)	NS	4.7 ± 8.7	5.0 ± 8.1	NS
Total red/orange complex species	8 (100)	8 (100)	NS	48.0 ± 29.2	24.6 ± 19.4	0.012
Other bacteria:						
* Aggregatibacter actinomycetemcomitans*	2 (25.0)	2 (25.0)	NS	0.02 ± 0.05	0.02 ± 0.04	NS
Spirochetes	6 (75.0)	4 (50.0)	NS	5.8 ± 4.9	6.8 ± 5.6	NS
Motile rods	8 (100)	7 (87.5)	NS	23.9 ± 12.0	24.1 ± 14.6	NS
Total motile morphotypes	8 (100)	7 (87.5)	NS	28.3 ± 15.0	24.5 ± 16.7	NS

Abbreviation: NS, not statistically significant.

## 4 DISCUSSION

A periodontal diagnosis would ideally infer the most likely prognosis and best‐practice therapy. For example, it would be important to diagnose progressive periodontitis which has a worse prognosis and requires a more comprehensive anti‐infective treatment than disease‐stable periodontitis. The present study explored whether periodontal pocket temperature differences would help identify sites which exhibited elevated levels of major periodontopathic bacteria with an increased presumed risk for periodontal breakdown (Rams et al., 1996).

The major finding of this pilot study was a significant relationship between subgingival *P. gingivalis* and elevated periodontal pocket temperature. A combination of other red/orange complex periodontal pathogens, besides *P. gingivalis*, was also significantly associated with elevated subgingival temperature. The microbial‒subgingival temperature relationships were robust enough to be statistically significant even with the relatively small number of paired intra‐patient comparisons. In contrast, bacteria with little or no periodontopathic potential elicited no measurable temperature increase. The intra‐patient microbiological comparisons between hot versus cool deep periodontal pockets with bleeding on probing have not been previously reported and strengthens the significance of the observed higher levels of periodontal pathogens in hot temperature sites.

The present findings are consistent with and expand upon prior reports associating hot periodontal sites with elevated levels of *P. gingivalis*, *P. intermedia/nigrescens*, *P. micra*, and *A. actinomycetemcomitans* and cool periodontal sites with health‐related *Capnocytophaga* species and viridans streptococci (Haffajee et al., 1992c; Rams & Slots, 2023). Dental implant sites show a similar microbial trend, with *Streptococcus* species predominating at cool peri‐implant sites (Rams et al., 1993b). However, the microbial correlation with subgingival temperature seems less clear in periodontal maintenance patients (Wolff et al., 1997).

It is not known if important periodontal pathogens caused the elevated subgingival temperatures or the elevated subgingival temperatures promoted upgrowth of periodontal pathogens, or both. Periodontal infection by *P. gingivalis* (Taylor, 2010) or active herpesvirus (Contreras et al., 2014) may trigger release of pro‐inflammatory cytokines and enzymes (Wolff et al., 1997) capable of raising periodontal tissue temperature. Reasons for upgrowth of *P*. *gingivalis* in hot periodontal pockets may be related to the species’ upregulation, at high temperature, of superoxide dismutase production (Amano et al., 1994) and enhanced organism binding to hemoglobin‐derived iron protoporphyrin IX (Smalley et al., 2000), both of which serve to protect against bacterial killing by neutrophils. Also, *P. gingivalis* and *F. nucleatum* produce butyric acid, which can activate latent herpesviruses (Imai & Ogata, 2020), with subsequent impairment of humoral immunity and upgrowth of *P. gingivalis* (Slots et al., 2003). Apparently, both host factors and bacteria‐herpesvirus interactions (Chen et al., 2020) may contribute to increases in subgingival temperature and major periodontopathic bacteria.

Of major interest, high subgingival temperatures may indicate an increased risk for periodontal breakdown (Haffajee et al., 1991, 1992b; Lindskog et al., 1994). Hot periodontal sites were more than twice as likely as cool periodontal sites to experience progressive periodontitis within a 2‐month period (Haffajee et al., 1992b). Most strikingly, shallow pockets (<4 mm) with elevated temperature and bleeding on probing experienced new attachment loss in 8.9% of sites, whereas shallow pockets with cool temperature and bleeding on probing demonstrated progressive attachment loss in only 1.4% of sites (Haffajee et al., 1992b). Also, periodontitis patients with elevated subgingival temperatures plus high levels of *P. gingivalis* or *Campylobacter rectus* showed significantly more disease‐active sites than patients with a combination of cool subgingival temperature and low periodontal pathogen levels (Haffajee et al., 1991). Since severe herpesvirus infection, like bacterial pathogens, is more prominent in disease‐active than in disease‐stable periodontitis sites (Kamma et al., 2001; Slots et al., 2003), and usually coexist with major periodontopathic bacteria (Chen et al., 2020; Slots & Rams, 2023), hot periodontal sites are also anticipated to harbor high herpesvirus copy counts with increased risk for periodontal breakdown.

## 5 CONCLUSIONS

Elevated periodontal pocket temperature was associated with significantly higher subgingival proportions of *P. gingivalis* and total subgingival red/orange complex species. This observation may contribute to a better understanding of periodontal disease pathogenesis and aid in a clinical setting to differentiate between progressive and nonprogressive periodontitis. Clinical trials are however needed to assess the utility of subgingival temperature to predict periodontitis disease activity and the efficacy of periodontal therapy. A more widespread acceptance of subgingival temperature diagnostics may also require improved thermocouple devices. A recently developed periodontal temperature probe includes wireless transfer of subgingival readings to interpretive computer software (Ritzert et al., 2022).

## AUTHOR CONTRIBUTIONS

Both authors made substantial contributions to the conception and design of the study, the acquisition of data, and the analysis and interpretation of the data. Both authors drafted the manuscript, revised it critically for important intellectual content, and give final approval of the version to be published. Both authors take public responsibility for the content of the manuscript and agree to be accountable for all aspects of the work.

## CONFLICT OF INTEREST STATEMENT

The authors declare no conflict of interest.

## ETHICS STATEMENT

The study was conducted in accordance with the Declaration of Helsinki, with approval of the Temple University Institutional Review Board, and the patient signed informed consent.

## Data Availability

Research data are not shared.
